# Correspondence

**Published:** 1868-04

**Authors:** D'Oyley Evans, P. H. Austen

**Affiliations:** Baltimore.


### CORRESPONDENCE.

---

BALTIMORE, *February*, 18, 1868.

PROFESSOR GORGAS,

*Dear Sir:*

THE accompanying letter, just received from Paris will be read by your subscribers with much interest. I am, at present, too much engrossed by my collegiate and other duties to make experiments with the new cement. Under these circumstances, Dr. Evans will, I trust, excuse me for making public his letter *before* "having experimented." His description is so clear and points to properties so valuable, that your readers will not need to wait upon any experiments by me, or any one else. Let every one make trial for himself and then let him make known the results. Dr. Evans is indefatigable in his search into the domains of Physical and Chemical Science, for what is practically useful to the dentist: and with a liberality characteristic of the true lover of science, he is anxious that all shall share as quickly as possible in whatever benefit may accrue from his researches.

---

2 PLACE DE LA TRINITE,
PARIS, *Jan.* 30, 1868.

DEAR DOCTOR:—

I promised to inform you of any thing new or interesting, that might attract my notice, applicable to mechanical dentistry. I take great pleasure in writing to you of a new CEMENT which was presented to the Academy of science by M. SOREL, a few weeks ago.

Experimenting with it, to ascertain whether it would not be a good substitute for plaster, in making models, I find it superior. The models are not only neater and more durable; but become almost as hard as Parian marble and quite as beautiful.

The substance used is a basic hydrated oxy-chloride of magnesium. It may be obtained by slaking magnesia in a solution of chloride of magnesium, more or less concentrated. The denser the solution, the harder it becomes on drying.

This magnesian cement is the whitest and hardest of any now known and can be moulded like plaster of Paris, acquiring, as already stated, the hardness of marble. It will take any color and has been used by M. SOREL for making mosaics, imitations of ivory, billiard balls, &c.

It possesses the *agglutinative* property in the highest degree, by virtue of which it forms solid mases, when mixed, in small proportion, with certain abundant and cheap substances. With twenty times its weight of sand limestone and other inert matters, it will form hard blocks; while lime and other cements will admit of only two or three times their weight. It promises to become extremely valuable as a building material, in localities where brick and stone are scarce or expensive.

The magnesian cement may be obtained at low cost, wherever the magnesia is extracted from the mother-ley of salt-works; either by M. Balard's process, which gives magnesia and hydro-chloric acid at the same time; or by decomposing the ley by quicklime; yielding, by double decomposition, magnesia and chloride of lime—containing a certain quantity of chloride of magnesium and forming with the addition of various cheap substances, a good white-wash.

If after experimenting with this cement, you also obtain favorable results, you are at liberty to publish in the Journal of Dental Science this letter or any part of it you may think proper.

Yours very truly,

DR. D'OYLEY EVANS.

Prof. P. H. Austen, Baltimore.

